# Research on Sea State Signal Recognition Based on Beluga Whale Optimization–Slope Entropy and One Dimensional–Convolutional Neural Network

**DOI:** 10.3390/s24051680

**Published:** 2024-03-05

**Authors:** Yuxing Li, Zhaoyu Gu, Xiumei Fan

**Affiliations:** 1 School of Automation and Information Engineering, Xi’an University of Technology, Xi’an 710048, China; 2 Shaanxi Key Laboratory of Complex System Control and Intelligent Information Processing, Xi’an University of Technology, Xi’an 710048, China

**Keywords:** sea state signal, slope entropy, beluga whale optimization, one-dimensional convolutional neural network, feature extraction

## Abstract

This study introduces a novel nonlinear dynamic analysis method, known as beluga whale optimization–slope entropy (BWO-SlEn), to address the challenge of recognizing sea state signals (SSSs) in complex marine environments. A method of underwater acoustic signal recognition based on BWO-SlEn and one-dimensional convolutional neural network (1D-CNN) is proposed. Firstly, particle swarm optimization–slope entropy (PSO-SlEn), BWO-SlEn, and Harris hawk optimization–slope entropy (HHO-SlEn) were used for feature extraction of noise signal and SSS. After 1D-CNN classification, BWO-SlEn were found to have the best recognition effect. Secondly, fuzzy entropy (FE), sample entropy (SE), permutation entropy (PE), and dispersion entropy (DE) were used to extract the signal features. After 1D-CNN classification, BWO-SlEn and 1D-CNN were found to have the highest recognition rate compared with them. Finally, compared with the other six recognition methods, the recognition rates of BWO-SlEn and 1D-CNN for the noise signal and SSS are at least 6% and 4.75% higher, respectively. Therefore, the BWO-SlEn and 1D-CNN recognition methods proposed in this paper are more effective in the application of SSS recognition.

## 1. Introduction

The earth is abundant in marine resources. With the advancements in human productivity and technology, there is a growing interest in utilizing, developing, and protecting the ocean [1,2,3,4]. In underwater signal transmission, acoustic signals serve as the primary carriers of information. However, underwater acoustic signals frequently exhibit non-stationary and nonlinear characteristics. Therefore, the key challenge lies in extracting information from non-stationary nonlinear signals [5,6]. Most traditional methods for extracting features from underwater acoustic signals involve converting the signals from the time domain to the frequency domain and extracting the spectral features. Short-time Fourier transform and Wigner–Ville distribution fail to adequately capture the relationship between signal frequency and time. Despite its ability to provide time–frequency information, the wavelet transform is limited by the choice of wavelet basis function [7,8]. While these methods have shown some success in practical applications, characterizing the nonlinearity of underwater acoustic signals remains challenging. Consequently, the traditional signal-processing methods are limited in their ability to address the nonlinear characteristics of underwater acoustic signals. Thus, there is a need to propose a new method for characterizing these nonlinear characteristics.

With the advancement of nonlinear science theory, fractal dimension (FD) has been proposed, but the traditional FD cannot represent the complexity information of multi-channel time series. Therefore, Yuxing Li et al. [9] proposed multivariate multiscale HFD (MvmHFD). And the concept of information entropy, which quantifies the complexity and randomness of signals, has been introduced. Entropy measures such as approximate entropy (AE) [10,11], SE [12,13], FE [14,15], PE [16,17], and DE [18,19] have found extensive applications in the field of underwater acoustic signal recognition. The scope of entropy measurement methods has expanded over time, giving rise to various types. For instance, Costa proposed multiscale entropy (MSE) [20,21], which assesses the complexity of time series across different scales. Building upon MSE, Jinde Zheng et al. introduced multiscale permutation entropy (MPE) [22,23], multiscale fuzzy entropy (MFE) [24,25], and adaptive multiscale dispersion entropy (AMDE) [26,27]. Based on SE and MSE, Keheng Zhu et al. presented a hierarchical entropy (HE) method [28]. This approach involves hierarchical data decomposition, using sample entropy at each decomposition node to construct feature vectors, which are then used for fault identification through support vector machine techniques. Yongbo Li et al. proposed hierarchical fuzzy entropy (HFE) [29], a hierarchical analysis combined with fuzzy entropy calculation. Unlike MFE, HFE analyzes both low-frequency and high-frequency signal components, enabling more comprehensive and accurate information extraction. Yuxing Li et al. [30] developed a variable-step multiscale KFD (VSMKFD) to extract ship radiated noise features.

In 2019, David CuestaFra proposed a novel time series complexity estimator known as slope entropy (SlEn) [31,32]. Due to its significant impact on classification, SlEn has been widely used in various fields. Its effectiveness has been verified in disciplines such as medicine, hydroacoustics, and fault diagnosis. For example, Dakappa Pradeepa H et al. [33] conducted a time series analysis of fever using SlEn, while CuestaFra David et al. [34] applied SlEn to classify activity records of bipolar disorder patients. In the field of underwater acoustic signal recognition, SlEn offers a comprehensive representation of the nonlinear characteristics of underwater acoustic signals, surpassing conventional entropy methods like Apen and SE. Furthermore, as SlEn incorporates two threshold parameters that influence its performance [35,36], Li Yuxing et al. [37] proposed the PSO-SlEn algorithm, which employs an optimization algorithm to determine these parameters. In the context of PSO, Li Wei et al. [38] introduced the concept of quantum behavior and proposed a variant called quantum-behaved particle swarm optimization (QPSO). The effectiveness of PSO was further demonstrated by Gao Yonglin et al. [39] in improving the performance of the LS-SVM model. This paper presents the BWO-SlEn algorithm for feature extraction in underwater acoustic signals.

With the development of machine-learning algorithms, we can classify the features of the extracted signals through 1D-CNN [40,41], and the effective features can be automatically learned through training, thus achieving better classification results. Therefore, the recognition method of BWO-SlEn and 1D-CNN is proposed in this paper and applied to underwater acoustic signal recognition.

The structure of this paper is divided into the following parts. Section 2 detail the algorithmic principle and steps of the proposed method, respectively. Section 3 and Section 4 demonstrate the recognition experiments and analysis of noises and SSS, respectively. Finally, in Section 5, the innovation and experimental conclusions of this paper are discussed.

## 2. Algorithms

### 2.1. Slope Entropy

SlEn is a complexity estimator for time series data that quantifies the complexity of a signal. It is a nonlinear dynamic indicator calculated solely based on the magnitude and five moments of the time series. A higher value of SlEn indicates a more complex signal, while a lower value suggests a simpler signal. The complete calculation procedure for SlEn is provided below:

Step 1: For a given time series, $D=\left\{d_{i},i=1,2,3,\ldots ,n\right\}$, where n indicates the number of elements in a time series. Then, set the embedding dimension, m. The time series, D, can be divided into k=n−m+1 subseries. The decomposition form of the time series, D, is as follows:

(1) $$D_{1}=\left\{d_{1},d_{2},\ldots ,d_{m}\right\},D_{2}=\left\{d_{2},d_{3},\ldots ,d_{m+1}\right\},\ldots ,D_{k}=\left\{d_{k},d_{k+1},\ldots ,d_{n}\right\}$$

Step 2: For all subsequences obtained in (1), subtract the former from the latter of the two adjacent elements to obtain k new sequences:

(2) $$T_{1}\left(i=1,2,\ldots ,k\right)=\left\{t_{k},t_{k+1},\ldots ,t_{n-1}\right\}$$

Each of the *k* new sequences in (2) contains m−1 elements, which are $t_{k}=d_{k+1}-d_{k}$.

**Figure 1:**
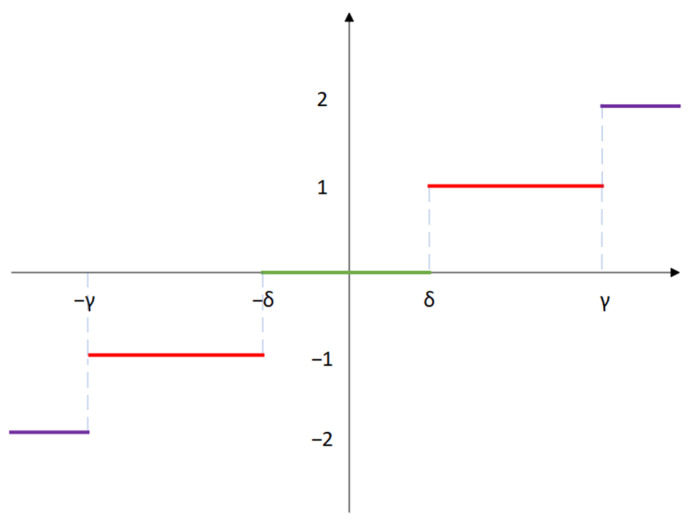
The principle of module division.

Step 3: Introduce the two SlEn threshold parameters, γ and δ. Here, 0<δ<γ. Using the threshold parameters −γ, −δ, δ, and γ as the dividing line, the numeric field is divided into five modules, namely −2, −1, 0, 1, and 2, and all elements in the sequence obtained from Step 2 are compared to these two thresholds. If $t_{k}<-\gamma$, it is divided into module −2. If $-\gamma <t_{k}<-\delta$, it is divided into module −1. If $\left|t_{k}\right|<\delta$, it is divided into module 0. If $\delta <t_{k}<\gamma$, it is divided into module 1. If $t_{k}>\gamma$, it is divided into module 2. The principle of module division is shown in Figure 1:

The partition sequence form is as follows:

(3) $$E_{k}=\left\{e_{k},e_{k+1},\ldots ,e_{n-1}\right\}$$

Each element of $E_{k}$ in (3) is one of the five modules, −2, −1, 0, 1, and 2.

Step 4: Since there are five modules, there are, at most, $j=5^{m-1}$ types of sequence $E_{k}$. For example, when m is 3, there are, at most, 25 types of $E_{k}$. These are {2,2}, {2,1}, {2,0}, {2,−1}, {2,−2}, …, {−2,−1}, and {−2,−2}; the number of each type is recorded as $r_{1},r_{2},\ldots ,r_{j}$, and the frequency of each type is calculated as follows:

(4) $$R_{j}=\frac{r_{j}}{k}$$

Step 5: Based on the classical Shannon entropy, the formula of SlEn is defined as follows:

(5) $$SlopeEn\left(m,\delta ,\gamma \right)=-\sum_{j}R_{j}\ln R_{j}$$

where $R_{j}$ is obtained from (4).

### 2.2. Beluga Whale Optimization

The BWO [42] algorithm is an optimization based on the bionics idea, which simulates the predation and mating behavior of the beluga whale population. It can be utilized for function optimization, machine learning, and neural networks. The following are the complete steps of the BWO algorithm:

(1) First, an appropriate optimization problem is selected and transformed into a mathematical model. The determination of whether the target necessitates a minimum or maximum value, as well as the identification of the optimization variables, is required.
(2) Initializing the beluga population. Specifically, beluga whale individuals are randomly generated, each containing optimization variables, which can be represented by a d-dimensional vector.
(3) The fitness value of each individual beluga is calculated. Specifically, the optimization variable of each individual is substituted into the objective function to obtain the corresponding fitness value.
(4) The beluga population members are ranked based on their fitness values. Specifically, the individual beluga whale with the highest fitness value is referred to as the “leader” individual.
(5) The positions and speeds of the remaining beluga whales are updated based on the position and speed information of the leader.
(6) The fitness value of the updated beluga whale population is recalculated, while the global historical optimal solution is updated.
(7) Determine whether the maximum number of iterations has been reached. If it is satisfied, the optimal solution is generated, and the algorithm ends. Otherwise, go back to Step 4 and continue the iteration.

### 2.3. Beluga Whale Optimization–Slope Entropy

BWO-SlEn is an improvement of SlEn, and its basic idea is to optimize the two threshold parameters of an SlEn through an optimization algorithm. According to this idea, the signal recognition methods of BWO-SlEn and 1D-CNN are proposed in this paper. The basic steps are as follows:

Step 1: The average recognition rate of 1D-CNN recognition signals is taken as the objective function, and the two independent variables of the function are δ, and γ.

Step 2: Algorithm initialization. Set the population quantity as “noop” and the maximum number of iterations as “T_max_”. Seek the values of δ and γ corresponding to the maximum average recognition rate.

Step 3: Calculate the fitness (average recognition rate) of each individual, find the individual with the highest fitness, and designate it as the “leader” individual.

Step 4: According to the position and speed information of the leader, the BWO algorithm is used to update the position and speed of other individuals.

Step 5: If the maximum number of iterations, T_max_, has not been reached, iterate once more from Step 3 in order to identify the individual displaying the highest level of fitness.

Step 6: After reaching the maximum number of iterations, T_max_, output the iteration time and the two parameter values represented by the individual with the highest fitness.

**Figure 2:**
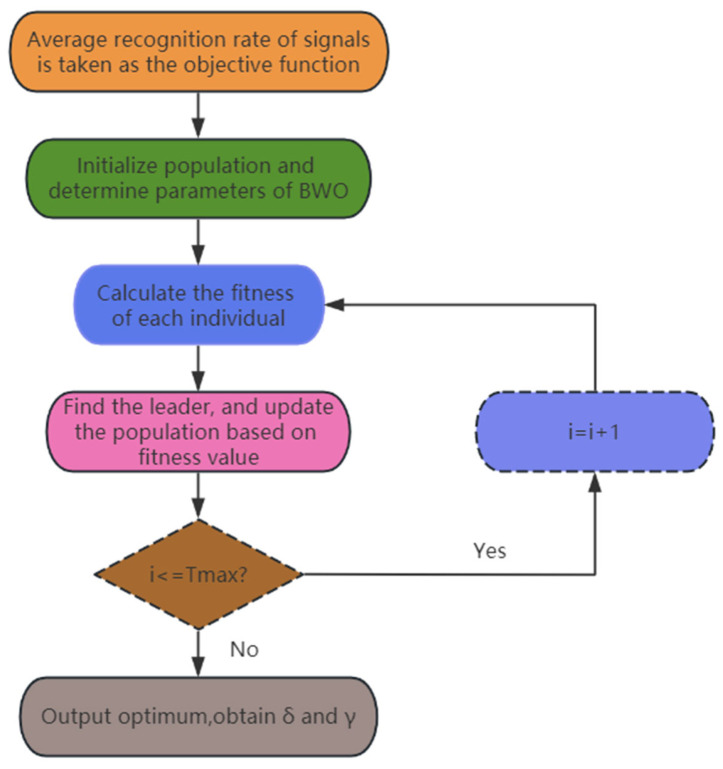
The flow of BWO-SlEn algorithm.

The flow of the BWO-SlEn algorithm is shown in Figure 2.

### 2.4. One Dimensional Convolutional Neural Network

**Figure 3:**
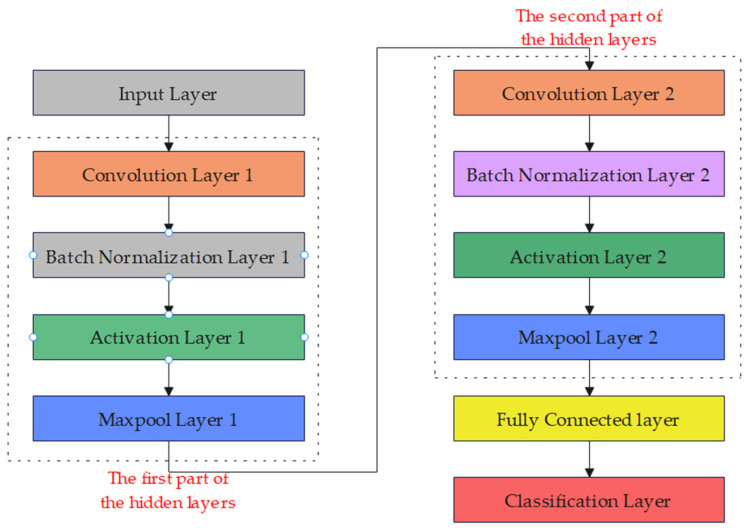
The 1D-CNN model structure.

A 1D-CNN [43] is a variant of the convolutional neural network. The 1D-CNN is mainly used to process one-dimensional sequence data. Compared with traditional fully connected neural networks, the 1D-CNN can better deal with local relationships in sequence data. The network structure used in this study is shown in Figure 3.

The dimension of the input data for the input layer is 15. The first part of the hidden layer is configured as follows: The convolutional layer consists of 64 convolutional kernels with a size of 2. The batch normalization layer performs batch normalization on the output of convolutional layer. The activation layer applies the rectified linear unit (ReLU) function to activate the output of the convolutional layer. The window size of the max pooling layer in the time dimension is 2, with a stride of 1. The second part of the hidden layer is much the same as the first, except convolutional layer 2 contains 32 convolutional kernels with a size of 2. The output size of the fully connected layer is 4, corresponding to 4 categories. The classification layer performs classification on the output and obtains the final prediction result.

The training parameters and configuration of the neural network are summarized as follows: the network parameters are updated by using the Adam gradient descent algorithm; a maximum of 500 epochs are conducted; the initial learning rate is set to 0.0001; L2 regularization parameter of 0.0001 is applied; the learning rate is decreased using a piecewise linear decay strategy, with the learning rate reduced to 0.1 times its original value after every 450 epochs; and before each epoch, the training set samples are shuffled.

### 2.5. The Recognition Method Based on Beluga Whale Optimization–Slope Entropy and One-Dimensional Convolutional Neural Network

Combining BWO-SlEn and 1D-CNN, a new SSS recognition method is proposed, BWO-SlEn is used for feature extraction, and 1D-CNN is used to classify the extracted features. The specific steps of the proposed method are as follows: 

Step 1: The SSSs are divided into signal samples for feature extraction. In this paper, four types of SSSs are divided into samples, and each SSS type has 3000 signal samples, with each sample containing 4096 data points.

Step 2: The signal samples of the SSSs are calculated by BWO-SlEn. In this paper, four types of SSS signal samples are calculated, each type of SSS to obtain 3000 feature samples, a total of 12,000 feature samples.

Step 3: The feature samples are input into the neural network for classification. In this paper, the 3000 feature samples of each type of SSS are reset to a 200 × 15 matrix and then input to 1D-CNN.

## 3. Noise Signal Classification and Recognition Based on Beluga Whale Optimization–Slope Entropy and One-Dimensional Convolutional Neural Network

### 3.1. Introduction to Noise Signal

**Figure 4:**
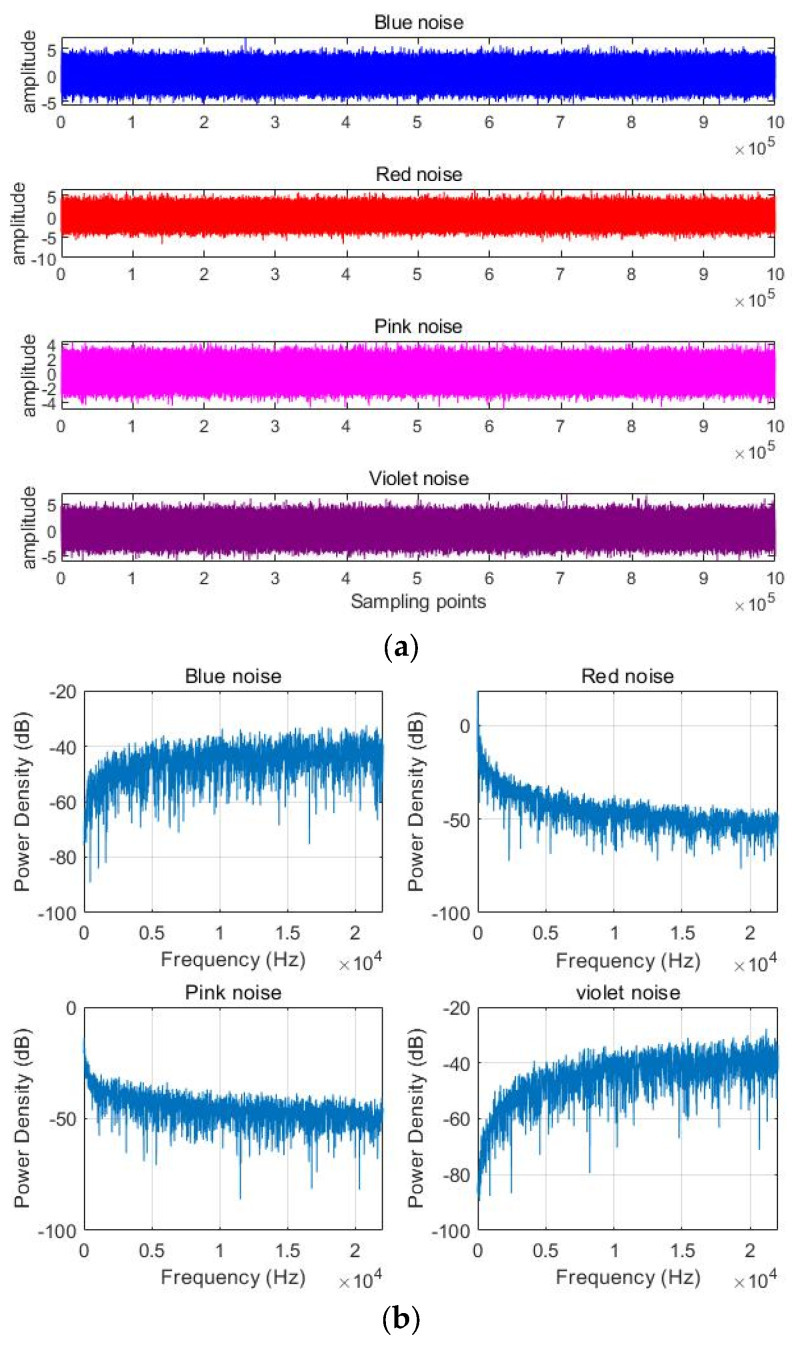
Noise signal waveforms in (**a**) time domain and (**b**) frequency domain.

The simulation signal uses four different noise signals, namely blue noise, red noise, pink noise, and violet noise. Blue noise can produce higher energy at higher frequencies, and its power density is proportional to frequency. Red noise can produce a lot of energy at low frequencies, and its power density is inversely proportional to each octave. The power spectral density of pink noise is inversely proportional to each octave, and the intensity of each octave is equal. Violet noise produces very high energy at higher frequencies, and its power density is proportional to each octave. The data length of each noise signal used in the experiment in this section is 16,384,000. Noise signal waveforms in the time and frequency domains are shown in Figure 4.

It can be seen from Figure 4 that, in the frequency domain, blue noise and violet noise are similar, and their power density is proportional to frequency. Red noise is similar to pink noise, and its power density is inversely proportional to frequency.

### 3.2. Slope Entropy Optimization

For the purpose of comparison, identical parameters are set for all three optimization algorithms. The parameter settings for these algorithms are presented in Table 1.

The three optimization algorithms undergo 30 iterations. In each iteration, the SlEn of all individuals in the population is computed, and then classification is performed using 1D-CNN. The objective function is set as the maximum recognition rate of the noise signal. The iteration time and the average recognition rate of the noise signal are then recorded. The optimization results of the three optimization algorithms are shown in Table 2.

Table 2 shows that, among the three optimization algorithms for optimizing SlEn, PSO-SlEn has the shortest optimization time but the lowest noise signal recognition rate. HHO-SlEn has the longest optimization time but a moderate recognition rate. However, BWO-SlEn has an intermediate optimization time and the highest recognition rate. Therefore, among the three optimization methods, the feature extraction method of BWO-SlEn is the most effective for recognizing noise signals.

### 3.3. Based on Different Feature Extraction and One-Dimensional Convolutional Neural Network

To demonstrate the effectiveness of the BWO-SlEn and 1D-CNN methods in noise signal recognition, seven signal recognition methods are compared in this experiment, including the unoptimized SE, FE, PE, DE, and 1D-CNN recognition methods, as well as the three optimized SlEn and 1D-CNN methods proposed in Section 3.2. The parameter settings for the unoptimized entropy method can be found in Table 3.

Starting from the first data point of the signal, the generated noise signal is sampled, with every 4096 data points being taken as a signal sample. A total of 3000 signal samples are obtained for each type of noise. The 3000 noise signal samples are calculated by using the entropy value to obtain 3000 entropy samples. Every 15 entropy samples are served as one neural network sample, resulting in 200 neural network samples for each type of noise. The neural network samples for each type of noise are divided into 100 samples for the test set and the remaining 100 samples for the training set.

Multiple entropy methods are employed to extract the complexity features from four types of noise. The dimensionality of the entropy sample’s feature points is 15. To facilitate visualization, T-Distributed Stochastic Neighbor Embedding (T-SNE) is utilized for reducing their dimensionality to a two-dimensional space. T-SNE map maps high-dimensional features to a two-dimensional space, with the horizontal and vertical coordinates being the two dimensions of the mapping, without any actual coordinates or meanings. 

**Figure 5:**
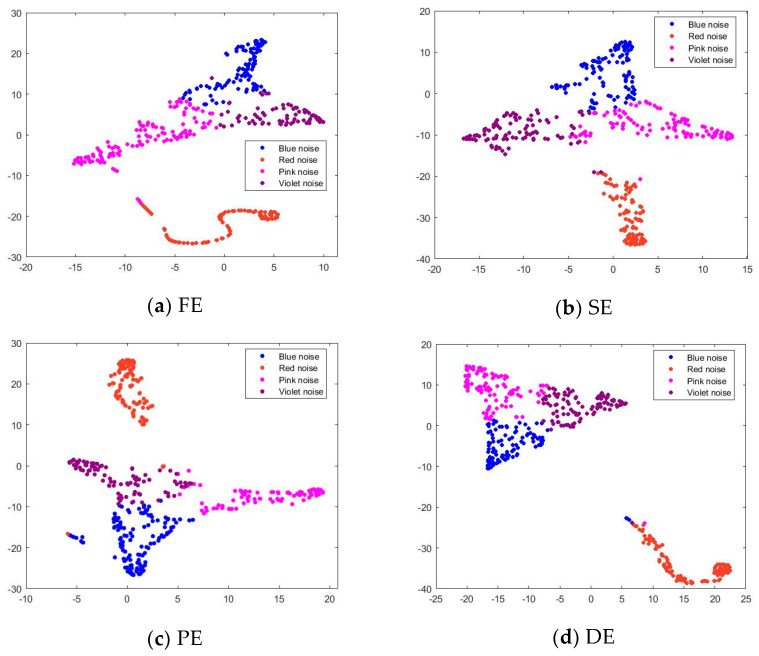

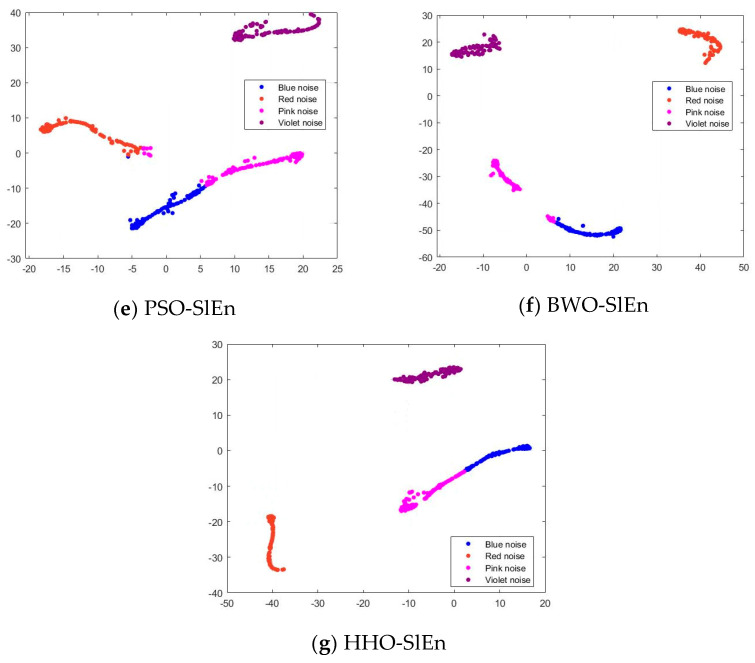
Two-dimensional scatterplot of output layer features of T-SNE test set based on different features.

In the two-dimensional descending scatter diagram of the output layer features of the T-SNE test set, different colors represent different signals. The relationship between signals in different feature extraction can be visually observed by the degree of aggregation of dimensionality reduction points. The denser the points of the same color are and the further away they are from other points of the same color, the more it indicates that the signal is recognized correctly after such feature extraction. If several different points gather together more, it can show that these signals are not easy to distinguish between each other after this feature extraction. The two-dimensional descending scatter diagram of the output layer features of the T-SNE test set based on different features is shown in Figure 5.

To demonstrate the effectiveness of BWO-SlEn, this study calculates the number of correctly recognized signals in each signal class and the overall signal recognition rate for each recognition method. The confusion matrix can quantify the classification situation of the signal through 1D-CNN based on the numbers after signal classification. Compared to Figure 5, it is much clearer to see the correct recognition rate for each signal.

**Figure 6:**
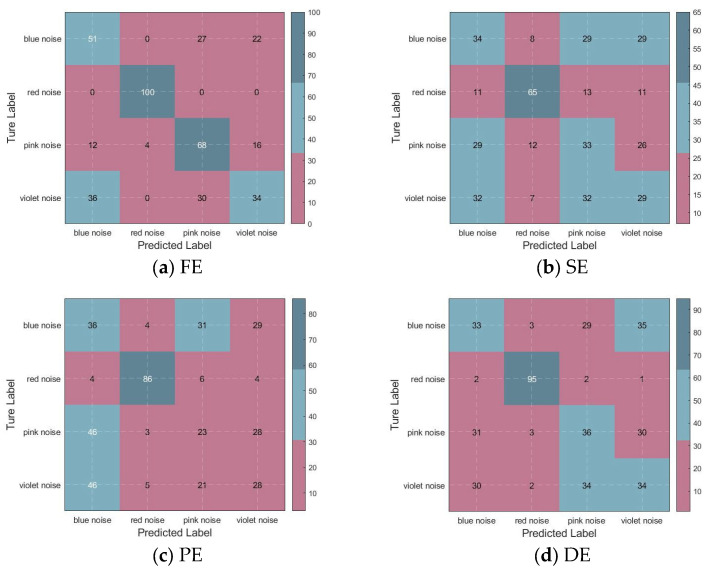

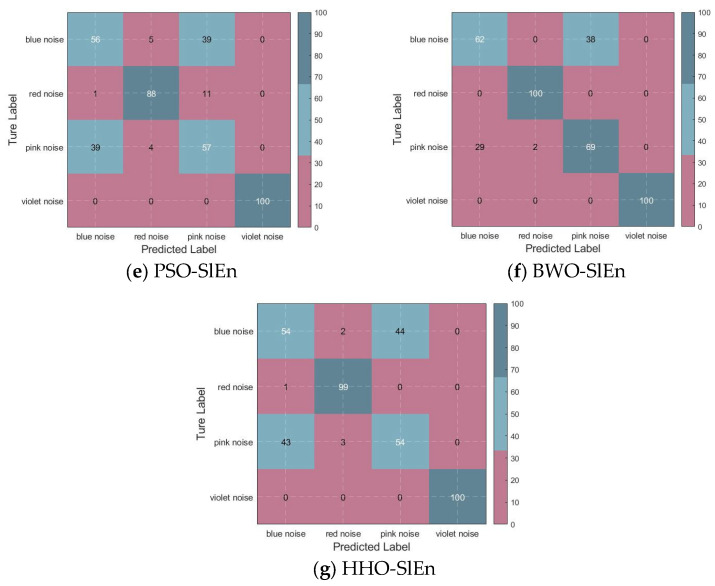
Confusion matrix for signal recognition.

The confusion matrix obtained after signal feature classification clearly shows the specific recognition situation of each type of signal, as shown in Figure 6. The recognition rate calculations are presented in Table 4 and are based on various feature extraction and 1D-CNN recognition methods for noise signal identification.

According to Figure 6 and Table 4, the recognition methods using FE and BWO-SlEn can completely identify the red noise; the three recognition methods using optimized SlEn can entirely identify the violet noise; the correct identification number of blue noise and pink noise for all recognition methods is below 70; the recognition rates of the recognition methods using optimized SlEn are higher than those of the unoptimized SlEn; among these three recognition methods using optimized SlEn, the recognition rates of BWO-SlEn are higher than others.

The experimental results demonstrate that the recognition methods using optimized SlEn have more effective recognition than unoptimized SlEn; among these three recognition methods using optimized SlEn, BWO-SlEn exhibits a better recognition ability than others. These demonstrate the necessity of optimizing SlEn parameters, the superiority of BWO global search, and the effectiveness of the method proposed in this paper for the accurate recognition of signals with nonlinear and non-stationary characteristics.

## 4. Experiments and Results

### 4.1. Introduction of Sea State Signal 

**Figure 7:**
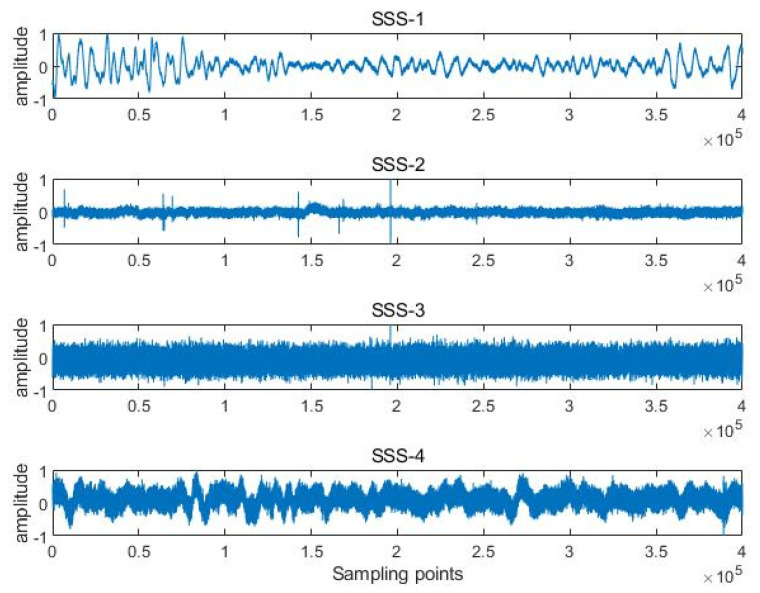
SSS waveforms in time domain.

Feature extraction is implemented for four types of measured SSSs, termed SSS-1, SSS-2, SSS-3, and SSS-4. Four types of SSSs derive from the same website [44]. The SSSs were collected from the Glacier Bay in Alaska, USA. SSS-1 represents light wind on the sea surface. SSS-2 represents snowfall on the sea surface. SSS-3 represents wind and ship noise. SSS-4 represents heavy rain on the sea surface. The sound of them was recorded by an underwater hydrophone. The length of the sampling point for SSS-1, SSS-2, and SSS-4 is 1,379,568, and the sampling length for SSS-3 is 1,313,904. The sampling frequency of them is 44,100 hz. SSS waveforms in the time domain are shown in Figure 7.

By studying and recognizing these signals, researchers can gain insights into the prevailing weather patterns, oceanic processes, and their impacts on marine ecosystems. This knowledge is crucial for maritime navigation, weather forecasting, climate studies, and marine resource management. It allows for the development of effective strategies to mitigate the adverse effects of these oceanic conditions on human activities and to promote safety and sustainability in marine environments.

### 4.2. Based on Different Feature Extraction and One-Dimensional Convolutional Neural Network

To demonstrate that the recognition effectiveness of SSS based on the BWO-SlEn and 1D-CNN method is the best, the experimental design steps for SSS are the same as those for noise signals. At the same time, the parameter settings for the optimization algorithms remain consistent with Table 1, and the parameter settings for the unoptimized entropy methods remain consistent with Table 3. The results of optimizing the SlEn using the three optimization algorithms for the SSS experiment are shown in Table 5.

As illustrated in Table 5, among the three optimization algorithms, the optimization time of HHO-SlEn is the longest, and the recognition rate of SSS is the lowest. PSO-SlEn has the shortest optimization time, but the recognition rate of sea is lower than that of BWO-SlEn. The optimization time of BWO-SlEn is moderate, and the recognition rate of SSS is the highest. Therefore, among the three optimization methods, the feature extraction method of BWO-SlEn is the most effective for SSS recognition.

**Figure 8:**
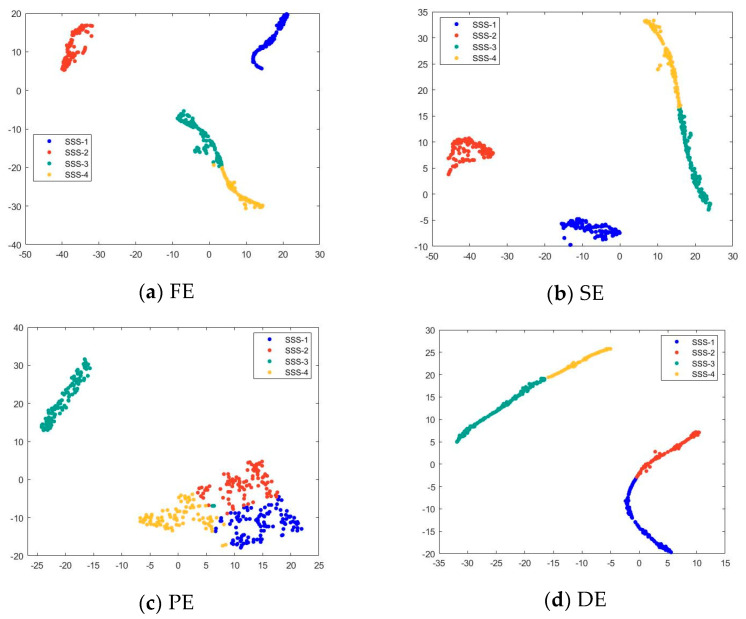

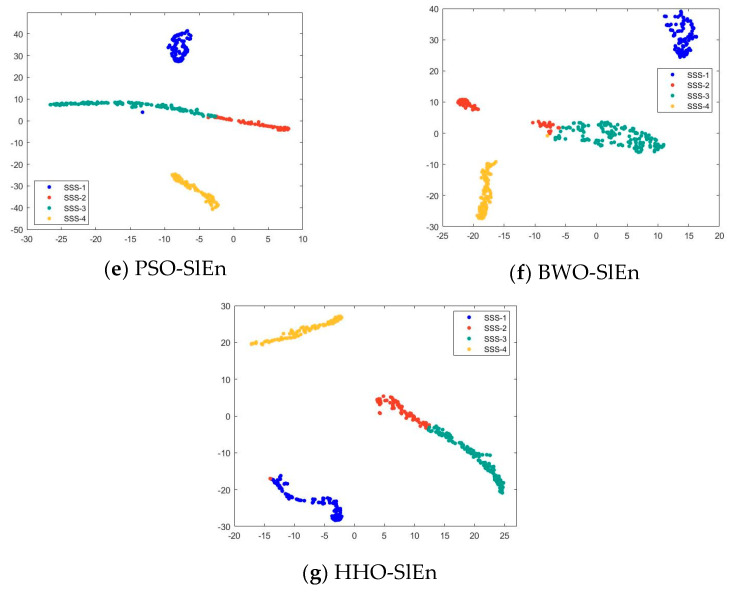
Two-dimensional scatterplot of output layer features of T-SNE test set based on different features.

Due to the limited length of the data of the SSS, in order to ensure the number of data points in the signal sample, when taking the data points of 3000 samples of the SSS sample, the starting point of the signal sample is randomly taken from the original signal, and then 4096 data points are taken backward. The starting point of SSS-1, SSS-2, and SSS-4 is set to the range of [1, 1,375,472], and the sample starting point of SSS-3 is set to the range of [1, 1,309,808]. A sample matrix of 3000 × 4096 is obtained for each SSS. The partition of the remaining entropy samples and neural samples is completely consistent with the simulation experimental signal. In order to facilitate observation, the dimension of the noise complexity feature points is reduced to two-dimensional space by T-SNE. The two-dimensional reduced-order scatter plot of the output layer features of the T-SNE test set based on different features is shown in Figure 8.

After performing a 1D-CNN classification on the four types of SSS features, the following information can be obtained from Figure 7. Among the recognition methods using unoptimized SlEn, PE only distinguishes SSS-3 well; DE has poor distinguishability for SSS-1 and SSS-2, as well as for SSS-3 and SSS-4; FE and SE exhibit low distinguishability for SSS-3 and SSS-4 but high distinguishability for the other two types of noise. Among the recognition methods using optimized SlEn, they show high distinguishability for SSS-1 and SSS-4, but PSO-SlEn and HHO-SlEn have lower distinguishability for SSS-2 and SSS-3 compared to BWO-SlEn.

**Figure 9:**
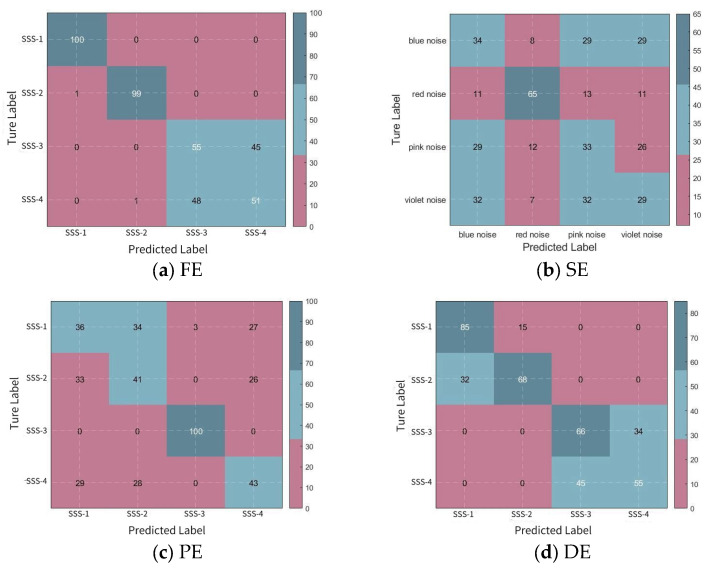

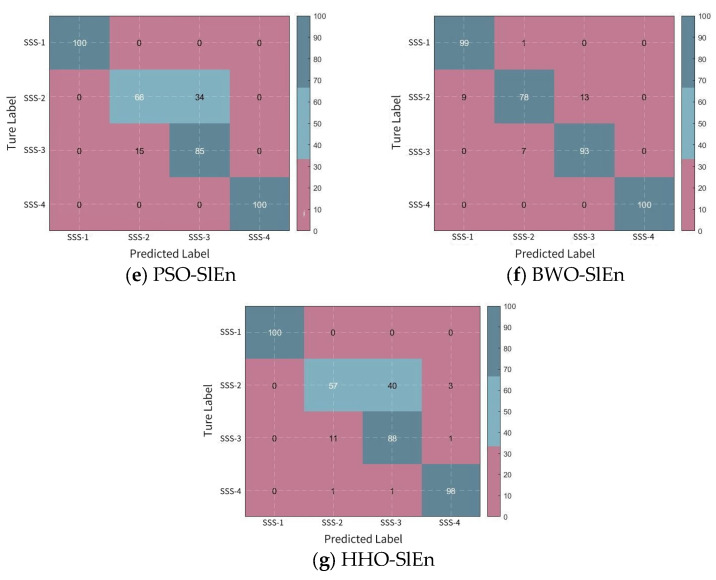
Confusion matrix for signal recognition.

To demonstrate the effectiveness of BWO-SlEn, this study calculates the number of correctly recognized signals in each signal class and the overall signal recognition rate for each recognition method. The confusion matrix obtained after signal feature classification clearly shows the specific recognition situation of each type of signal, as shown in Figure 9. The recognition rate calculations are presented in Table 6 and are based on various feature extraction and 1D-CNN recognition methods for noise signal identification.

As illustrated in Figure 9 and Table 6, the recognition methods using FE can recognize SSS-1 and SSS-2 above 99; the recognition methods using PE can completely identify SSS-3; the recognition methods that optimized SlEn can identify SSS-1 and SSS-4 above 98, and the recognition rates of them are higher than those of unoptimized SlEn; among the three recognition methods using optimized SlEn, only BWO-SlEn identified all four types of SSS above 75, and the recognition rate of BWO-SlEn is higher than others.

The experimental results demonstrate that the recognition methods using optimized SlEn have more effective recognition than unoptimized SlEn; among these three recognition methods using optimized SlEn, BWO-SlEn exhibits better recognition ability than others. This demonstrates that optimizing SlEn parameters is necessary; BWO possesses stronger global search capabilities in contrast to PSO and HHO, and the method proposed in this paper is effective for accurate recognition of SSS with nonlinear and non-stationary characteristics. This finding is in accordance with the results of our noise experiment.

## 5. Conclusions

Based on the theory of SlEn, the optimized SlEn is proposed and applied to the feature extraction of SSS, and then an SSS recognition method based on BWO-SlEn and 1D-CNN is proposed. The feasibility of the proposed method is verified through the experiment of classification and recognition of four types of noise signals and four types of SSS. The main innovation points and conclusions of this study are as follows:

(1) BWO was used to optimize SlEn for the first time, and this method was used to extract the features of SSS. Then, 1D-CNN was used as the classifier, and the signal recognition method of BWO-SLEN and 1D-CNN was proposed.
(2) Compared to the PSO-SlEn and HHO-SlEn methods, the BWO-SlEn and 1D-CNN method achieved at least 6% and 4.75% higher average recognition rates for noise signals and sea condition signals.
(3) The recognition rates of the proposed BWO-SlEn were 24.7% and 17.5% higher than the average recognition rates of the other six recognition methods in noise signal and SSS, respectively.

## Figures and Tables

**Table 1:** Parameter settings for different optimization algorithms.

Optimization Algorithms	Parameter Setting	
	Initial Population	Number of Iterations
PSO	10	30
BWO	10	30
HHO	10	30

**Table 2:** The optimization results of the three optimization algorithms.

Optimization Algorithm	Optimization Result			
	γ	δ	Optimization Time (s)	Recognition Rate (%)
PSO	0.4278	0.0001	81,231.45	75.25
BWO	0.2841	0.0495	121,458.37	82.75
HHO	0.1169	0.0166	168,911.81	76.75

**Table 3:** The parameter settings for the unoptimized entropy method.

Entropy Methods	Optimization Result			
	Embedding Dimension (m)	Threshold (r)	Number of Classes (nc)	Delay Time (tau)
SE	2	0.15	-	1
FE	2	0.15	-	1
PE	4	-	-	1
DE	4	-	6	1

**Table 4:** Four types of noise recognition results.

Feature Extraction	Number of Samples Correctly Identified				Average Recognition Rates (%)
	Blue Noise	Red Noise	Pink Noise	Violet Noise	
FE	51	100	68	34	63.25
SE	34	65	33	29	40.25
PE	36	86	23	28	43.25
DE	33	95	36	34	49.50
PSO-SlEn	56	88	57	100	75.25
BWO-SlEn	62	100	69	100	82.75
HHO-SlEn	54	99	54	100	76.75

**Table 5:** The results of SlEn optimization by different optimization algorithms.

Optimization Algorithm	Optimization Result			
	γ	δ	Optimization Time (s)	Recognition Rate (%)
PSO	0.83294	0.007075	109,758.06	87.75
BWO	0.4219	0.0283	264,082.98	92.50
HHO	0.1024	0.0226	296,172.29	85.75

**Table 6:** Four types of sea state signal-recognition results.

Feature Extraction	Number of Samples Correctly Identified				Average Recognition Rates (%)
	SSS-1	SSS-2	SSS-3	SSS-4	
FE	100	99	55	51	76.25
SE	100	100	63	45	77.00
PE	36	41	100	43	55.00
DE	85	68	66	55	68.50
PSO-SlEn	100	66	85	100	87.75
BWO-SlEn	99	78	93	100	92.50
HHO-SlEn	100	57	88	98	85.75

## Data Availability

The datasets analyzed during the current study are available from the corresponding author upon reasonable request.

## Nomenclature

FE	fuzzy entropy
SE	sample entropy
PE	permutation entropy
DE	dispersion entropy
AE	approximate entropy
MSE	multiscale entropy
MPE	multiscale permutation entropy
MFE	multiscale fuzzy entropy
made	adaptive multiscale dispersion entropy
HE	hierarchical entropy
HFE	hierarchical fuzzy entropy
T-SNE	T-Distributed Stochastic Neighbor Embedding
SSS	sea state signal
MvmHFD	multivariate multiscale HFD
QPSO	quantum-behaved particle swarm optimization
PSO	particle swarm optimization
BWO	beluga whale optimization
HHO	Harris hawk optimization
SlEn	slope entropy
1D-CNN	one-dimensional convolutional neural network
